# Parent Training Tailored to Parents With ADHD: Development of the Improving Parenting Skills Adult ADHD (IPSA) Program

**DOI:** 10.1177/10870547231217090

**Published:** 2023-12-28

**Authors:** Therese Lindström, Sofia Buddgård, Lena Westholm, Martin Forster, Sven Bölte, Tatja Hirvikoski

**Affiliations:** 1 Karolinska Institutet, Stockholm, Sweden; 2 Stockholm Health Care Services, Region Stockholm, Sweden; 3 Curtin University, Perth, Western Australia

**Keywords:** parental ADHD, parent training, parenting, parent-child relationships, behavioral problems

## Abstract

**Objective:**

To describe the development of the Improving Parenting Skills Adult ADHD (IPSA) parent training (PT) program, designed for parents with ADHD.

**Method:**

IPSA was developed using an iterative co-creation approach, involving parents with ADHD from the initial knowledge mobilization phase onwards. The program prototype was evaluated by 16 parents with ADHD, in an open trial of program feasibility.

**Results:**

IPSA was deemed feasible in terms of acceptability and levels of active participation, with no evidence of unintended harm. All but one participant completed the program, attending on average 84% of sessions. Pre-to-post within-group comparisons of targeted skills and outcomes revealed changes in the expected direction regarding, for example, use of introduced parenting skills (Cohen’s *d* = 1.3).

**Conclusion:**

The program prototype was found acceptable, accessible, and safe. Findings support the potential value of adapting PT protocols for parents with ADHD and warrant further evaluation of IPSA in a randomized controlled trial.

Attention-deficit/Hyperactivity Disorder (ADHD) is a neurodevelopmental condition (NDC) that is characterized by a persistent pattern of inattention and/or hyperactivity-impulsivity (American Psychiatric Association, 2013) and associated with difficulties in executive function, causing impairment in significant domains of life. Symptom profiles, needs, and skills vary widely among adults with ADHD (Mahdi et al., 2018). Of those raising a child, many report difficulties in parenting (Johnston et al., 2012; Park et al., 2017). In families where both parent(s) and child(ren) have ADHD, additional child-rearing challenges can arise (Deater-Deckard, 2017)—and make access to effective parenting interventions particularly important. However, although parental ADHD symptomatology has been associated with less favorable outcomes following conventional parent training (PT, (Chronis-Tuscano et al., 2017)), there remains a shortage of PT protocols specifically adjusted for parents who themselves have ADHD. In this paper, we describe the development of a PT program tailored for parents with ADHD, including an early-stage evaluation of the feasibility of the program prototype.

Many parents with ADHD face a combination of practical, psychosocial, psychiatric and parenting challenges (Johnston et al., 2012; McGough et al., 2005). Parental ADHD symptoms have been associated with more ineffective and harsh parenting behaviors (Park et al., 2017), and more household disorganization (Mokrova et al., 2010); both factors that can compromise parent-child interactions and influence children’s psychosocial outcomes (Marsh et al., 2020; Pinquart, 2017). The situation is often further complicated by the increased risk of externalizing behaviors in children with ADHD (Connor et al., 2010). Compared to neurotypical parents, parents with ADHD report more family conflict (Biederman et al., 2002), lower parental self-efficacy, and higher parenting stress (Williamson & Johnston, 2019). Given this accumulation of challenges and risk factors, their access to effective parenting support is imperative.

There is ample evidence for the effectiveness of PT in supporting parents in developing their parenting skills, improving parental self-efficacy (Weber et al., 2019), and reducing children’s externalizing behavioral problems (Daley et al., 2018; Mingebach et al., 2018). However, the success of PT interventions is commonly attenuated in families of parents who themselves report clinical-level ADHD symptomatology (Chronis-Tuscano et al., 2017).

Like parenting, PT put great demands on executive functions that are often challenged in adults with ADHD, including the ability to translate knowledge into goal-directed action and to regulate one’s own emotions or motivation (Crandall et al., 2015; Johnston et al., 2012). Parental ADHD symptomatology may further interfere with PT implementation for example by making it difficult to organize PT participation or practice newly acquired skills between sessions (Friedman et al., 2020; Johnston et al., 2012). The likelihood of dropping out of PT may be influenced by stigma, feelings of “not fitting in” with the group, and experiences of having challenges that differ from those of neurotypical participants (Smith et al., 2015). In addition, adult ADHD is associated with a number of factors (e.g., socioeconomic disadvantage and depression) known to increase the risk of low PT attendance, premature discontinuation, and reduced outcomes (Chacko et al., 2016; Nock & Ferriter, 2005). Meanwhile, it appears that pharmacological treatment of parental ADHD has only limited effects on parenting (Chronis-Tuscano et al., 2020) and does not significantly improve the efficacy of PT (Jans et al., 2015). Despite this, we are not aware of any study that has investigated whether it is feasible to tailor the PT protocol itself, to better respond to the needs of parents with ADHD.

The cognitive accessibility of a PT intervention has implications for its feasibility (e.g., acceptability, demands put on parents to engage actively, and safety) and effects on targeted outcomes. According to findings and ideas put forward by the scientific community (Table 1), parents with ADHD would benefit from an individualized and flexible PT protocol, including elements that target needs related to parental ADHD and difficulties with executive functioning and emotion regulation (e.g., Chronis-Tuscano et al., 2017; Crandall et al., 2015; Johnston et al., 2012). Examples of potentially beneficial PT adaptations include measures to use inclusive language, facilitate active participation (e.g., attendance or between-session skill practice), and address parent’s own needs and prerequisites to effect change (e.g., improving household organization, developing compensatory strategies, or considering parental stress). Achieving this would likely require modifications of both content and delivery (Johnston et al., 2012).

**Table 1.** Examples of Findings, Ideas and Suggestions Concerned With Modifications and Adaptations of PT Likely to be Helpful for Parents With ADHD—Described and Put Forth by the Scientific Community^a^ and Applied in the IPSA Program.

Overall
- Recognize that adult ADHD symptoms can make it difficult to translate knowledge and plans into action, As well as to organize, actively participate in, and independently implement complex interventions - Address parental ADHD symptoms and related difficulties with executive functioning and emotion regulation - Address and attend to parents’ own needs first, for example, factors that can affect parents’ day-to-day functioning and prerequisites to implement what is learned in PT (e.g., stress, fatigue, nutrition, co-occurring conditions)
Content
- Incorporate components to target ADHD-related difficulties in parenting - Explicitly address parental organizational and planning skills (e.g., to reduce household disorganization) - Address parents’ use of compensatory strategies and own emotion regulation strategies - Provide support with problem solving, motivation, or low parental self-efficacy, when needed
Format
- Increase treatment dose; give room for repetition - Use a “hands-on,” skills-based approach - Reduce ADHD-related (e.g., executive, attentional) barriers to treatment adherence and skill implementation - Use an individually tailored, flexible protocol, for example including opportunities to tailor the pace, introduce skills more slowly, or allow for joint problem solving before introducing the next parenting skill - Provide regular therapist feedback to reinforce parenting efforts, point out small or subtle changes, strengthen the perception of progress, help parents “stay on track”, highlight/address implementation issues - Use short instructions, real-life examples and interactive or fast-paced learning (e.g., media presentations) - Allow parents to share successful strategies and solutions, normalize experiences - Include maintenance or booster sessions
Other
- Allow parents to make their own decision about starting PT - Keep enrolment and procedures simple, straight-forward, consistent - Prepare PT participation by addressing practical barriers to treatment engagement, ensuring realistic treatment expectations, and clarifying the role of the parent and therapist within the context of success - Engage therapists with good knowledge of ADHD - Establish good parent-therapist relationships, using a non-judgmental, caring and “equal level” approach - Consider incorporating components of CBT or mindfulness training

*Note*. CBT = cognitive behavioral therapy; PT = parent training.

a References: (Chacko et al., 2016; Chronis-Tuscano et al., 2017; Crandall et al., 2015; Friedman et al., 2020; Johnston et al., 2012; Nock & Ferriter, 2005; Park et al., 2017; Smith et al., 2015).

The process of developing behavioral and educational interventions might be described as an iterative circle of concept development, prototype evaluation, and program refinement (O’Cathain et al., 2019). Ideally, key stakeholders (e.g., representatives of future target participants) are involved as co-creators from early stages of knowledge mobilization onwards, for example, to help understand needs, identify priorities, and find acceptable solutions (O’Cathain et al., 2019). Decisions to move from the iterative intervention development phase to further evaluation in research are preferably based on early-stage assessments of whether the concept or prototype has the potential to achieve its intended functions (Elliott, 2021).

In this paper, we describe the development of the *Improving Parenting Skills Adult ADHD* (IPSA) PT program and report findings from an initial evaluation of its prototype. To identify needs for further program improvements and determine whether further research evaluation of IPSA is warranted, we assessed the feasibility (i.e., acceptability, levels of active participation, and safety) of the program and explored its preliminary effects on targeted skills and outcomes.

## Method

### Setting

IPSA was developed at the Center of Neurodevelopmental Disorders at Karolinska Institutet (KIND), Karolinska Institutet, Sweden, using a co-creation approach. The subsequent evaluation and iterative refinement of the program prototype was carried out in collaboration with the ADHD Center, Habilitation & Health, Region Stockholm, Sweden. The publicly funded Habilitation & Health provides outpatient habilitation services to individuals with disabilities. The ADHD Center is an outpatient unit offering psychoeducational interventions on ADHD.

### Intervention Development

The IPSA program was developed in three phases (Supplemental Table S1), the first two of which were carried out on behalf of the Swedish Board of Health and Welfare. First (phase 1), we compiled a report on parenting interventions in the context of parental ADHD (Hirvikoski, Lindström, Nordin, Jonsson, & Bölte, 2017). By interviewing stakeholders (*n* = 14 parents with ADHD and *n* = 20 professionals in relevant fields), and conducting a systematic literature review, we were able to integrate evidence from research with clinical practice expertise and knowledge of parents’ circumstances, experiences, and preferences. Next (phase 2), we developed the program concept and prototype. This was done based on workshops with parents with ADHD (*n* = 4), an anonymous survey about valued aspects of parenting (*n* = 38 parents with ADHD), further consultations with parenting support professionals (*n* = 2), and literature of relevance. Finally (phase 3), we put the program prototype to test in a small study of its feasibility (described below).

Parents with ADHD were involved throughout the program development process—in the generation of ideas about content and delivery (interviewees and survey responders in phases 1 and 2), and in the iterative circle of prototype evaluation and refinement (IPSA participants in phase 3). This co-creation approach was decisive to the design of the program, not least by emphasizing the importance of offering support in relation to parents’ reported own needs, prerequisites, and strategies for managing practical aspects of everyday family life in parallel with efforts to facilitate the implementation of introduced parenting skills.

### The Intervention

IPSA is a manualized PT program for parents with ADHD who have a child aged 3 to 11 years. The program is based on and incorporates key components and parenting skills from evidence-based PT protocols (e.g., (Barkley, 1997; Kling et al., 2010). However, adaptations have been made to increase its acceptability and accessibility for parents with ADHD (see Table 1 for examples). In essence, it alternates between structured group sessions that introduce parenting skills and individualized support to facilitate their implementation at home.

#### Program Principles

IPSA uses an *ipsative* perspective, where development takes place and is evaluated in relation to the target individual’s starting position and individual goals (i.e., not in relation to certain norms). The program combines a behavioral and an occupational therapy perspective: it uses a client-centered, resource-oriented, and solution-focused approach, focusing on needs rather than difficulties, building on what works and relying on participant (patient) expertise. It frames parenting as a set of skills that can be developed and centers the work on behaviors that can be increased or decreased to approach goals. It addresses parental activities in relation to their everyday context, seeks to concretize how the introduced parenting skills can be applied behaviorally, and emphasizes that even small steps and minor changes can make a difference.

#### Program Structure, Content, and Materials

IPSA is delivered in closed groups of up to nine parents by two group leaders: an occupational therapist and a psychologist (or professional with similar training) with relevant experience. The program consists of 14 weekly sessions, plus a booster group session. It begins with an individual intake session, whereafter parents participate in group sessions every 2 weeks (six occasions in total) and meet individually with the occupational therapist on the weeks in between (six occasions in total), before the individual closing session (Supplemental Table S2).

**Figure 1.**
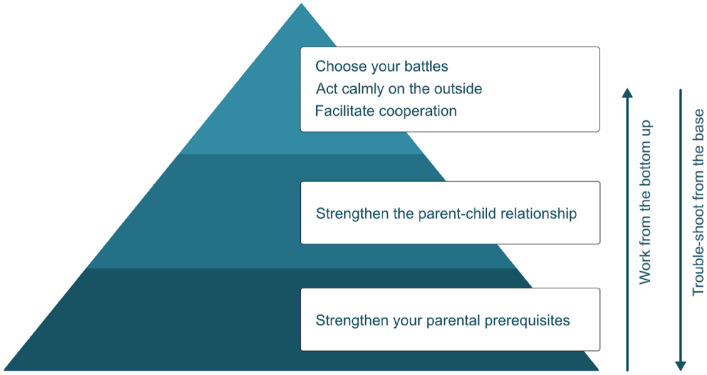
Illustration of how the IPSA program, content-wise, is structured like a pyramid where each part (focus, theme or skill) builds on the previous one(s). *Note*. Basically, parents work their way from the bottom up, putting special efforts into developing strategies for strengthening their own prerequisites (the base) and to improve the parent-child relationship (the middle), whereafter they gradually add skills to improve cooperation and reduce the risk of conflict (the top). One group session (number 5) is devoted entirely to parental emotion regulation and related skills. The pyramid analogy can also be used as a trouble-shooting tool, in such a way that parents who find themselves in a more challenging period (e.g., experiencing an increasing number of parent-child conflicts), are encouraged to check whether the base (their current prerequisites) or the middle (the relationship) are sufficiently stable—or need to be strengthened/tended to, before proceeding or returning to the top part of the pyramid.

In terms of content, IPSA is structured hierarchically like a pyramid, where each part (theme or skill) builds on the previous (Figure 1). Group sessions cover topics and skills such as: the manifestation and impact of parental ADHD in everyday family life; how to strengthen one’s own prerequisites for managing challenging everyday parent-child interaction situations; positive reinforcement and how to strengthen the parent-child relationship; effective prompts and how to facilitate child cooperation; how to reduce the risk of parent-child conflict and regulate one’s own emotional expression (Supplemental Table S2). During their between-session skill-training, parents focus on only *one* child (their “IPSA target child”) and center their work around *one* particularly challenging parent-child interaction situation (their “IPSA situation”).

The individualized support is largely offered by the occupational therapist (expert in helping individuals to enhance their ability to engage in desired everyday activities (Adamou et al., 2021)) and can be used by parents to overcome treatment barriers, strengthen their own prerequisites to use the introduced parenting skills, or problem-solve. If necessary to enable skills implementation, the occupational therapist and parent can agree to also address other factors that affect parents’ daily functioning, parenting or family function, such as household (dis-)organization, family routines, stress, or parental use of compensatory strategies.

The design of the program material was aimed at achieving high cognitive accessibility. Group sessions follow slide presentations and vary between short lecture segments, video skill demonstrations, and information processing components (e.g., group discussions). Parents are offered individualized appointment reminders and can access materials online. Cognitive aids (e.g., time-timers) and visual support are used as needed. Sandwiches are offered at group occasions.

### Study Design and Procedures

Participants in this uncontrolled pre-post treatment design study were self-referred parents with ADHD. Information about the project was disseminated through brochures and posters at the ADHD Center, through social media, during conferences, and other means of communication. Parents could register their interest in participating via the project website and were contacted in turn, based on the date of their application. After screening procedures (a structured interview by telephone), potential participants were invited to a complementary clinical assessment. To be eligible for participation, parents needed to have: an ADHD diagnosis (any presentation) confirmed in an assessment report or equivalent; at least one child (with or without an NDC) aged 3 to 11 years; sufficient knowledge of Swedish; the availability to participate in IPSA during the entire study period. The exclusion criteria included: diagnosed autism or intellectual disability; severe psychiatric conditions requiring urgent treatment (e.g., severe depression, suicidality, psychosis, or substance use disorder); major psychosocial difficulties or crisis in the family that necessitates the prioritization of other types of interventions. A total of 24 parents were contacted, 3 of whom declined participation and 5 of whom were excluded (4 had diagnosed autism, 1 had no child aged 3 to 11 years). Ultimately, 16 parents with ADHD were deemed eligible and included for participation in the study.

The intervention was delivered by an occupational therapist and one of two psychologists (all authors of this article, SBu, LW, TL). The first IPSA group contributed valuable feedback, based on which the program was refined before the second group started. Specifically, an additional occupational therapist session was added before the first group session, and refinements were made to the program material.

The study was approved by the Regional Ethics Committee of Stockholm, Sweden (dnr. 2017/2435-31/5) and conducted from January 2018 to January 2019. Participants received both written and oral information before giving their written consent.

### Measures

Details about the self- and parent-report scales used (e.g., response scales, range, and Cronbach’s alphas in the current sample) can be found in Supplemental Table S3.

#### Background and Demographic Variables

Parents answered a set of questions about sociodemographic factors and their IPSA target child. Parents’ clinical ADHD diagnoses were confirmed via medical records, assessment reports, or equivalent, and they rated their current ADHD symptomatology on the short 6-item version of the Adult ADHD Self-Report Scale, the ASRS screener (Kessler et al., 2005).

#### Measures of Acceptability

Overall treatment satisfaction (i.e., satisfaction with the IPSA program as a whole) was assessed using the Evaluation Questionnaire (EQ; (Bramham et al., 2009), modified to fit the PT context. When averaging ratings across items, a summary mean score of ≥3 (on a scale from 0 to 4) was interpreted as a positive evaluation, indicating satisfactory acceptability. The scale was supplemented with questions about whether IPSA was recommendable, a summative grade, and open-ended (free text) questions about ways in which the program had been good/helpful or could be made better or more helpful (Hesslinger et al., 2002). To detect any need for further development, the first IPSA group could supplement or deepen their free-text answers orally. Treatment satisfaction was also measured for each separate IPSA session, administering a subset of the EQ items at the end of each session. Treatment credibility was assessed using the Credibility/Expectancy Questionnaire (C/EQ; (Devilly & Borkovec, 2000; Nock et al., 2007), modified to fit the PT context.

#### Measures of Active Participation

Attendance was monitored continuously. Program completion rates were assessed based on the percentage of program starters who attended at least nine regular IPSA sessions (i.e., 9 of 13 sessions for the first IPSA group, 9 of 14 for the second IPSA group). Participants’ use of the introduced parenting skills was measured with a set of questions about the recent frequency of targeted parent behaviors, summed into a Skills use total score. The completion of between-session assignments was assessed using the first 2 items of the Homework Rating Scale (Kazantzis et al., 2004), modified to fit the PT context. Ratings of *how much* of the (homework) assignment parents had completed (i.e., quantity) and *how well* it had worked (i.e., quality) were averaged across sessions. As part of the program’s follow-up procedures, parents rated their *performance* in, and their *satisfaction* with, their way of managing their IPSA situation.

#### Measures of Safety

Potential harm was monitored by documenting and reviewing spontaneously reported adverse events (e.g., any unfavorable, potentially negative, event that occurred during the study period) and serious adverse events (e.g., threatening life or function, requiring hospitalization). To detect any deterioration with regard to parental mental health, participants rated their levels of general perceived stress, anxiety and depression before and after IPSA. This was done using the 10-item Perceived Stress Scale (PSS-10; (Cohen et al., 1983) and the Hospital Anxiety and Depression Scale (HADS; (Zigmond & Snaith, 1983).

#### Measures of Preliminary Effect-Related Outcomes

Effect-related outcomes were administered before (pre) and immediately after (post) IPSA.

Parental self-efficacy and participants’ perceptions of their own parenting was measured with the Parental Self-Efficacy scale (PSE; (Ulfsdotter et al., 2014). Parenting stress was measured with a Swedish version of the Parental Stress Scale (PSS; (Berry & Jones, 1995). Household disorganization (or the degree to which the home environment is perceived as chaotic, cluttered, disorganized, or hurried) was measured with the Confusion, Hubbub, and Order Scale (CHAOS; (Matheny et al., 1995), translated into Swedish using a standard translation-back translation procedure. The occurrence of behavior problems in the participants’ target children was measured with the Eyberg Child Behavior Inventory (ECBI; (Eyberg & Ross, 1978), which includes an Intensity Scale reflecting the frequency of 36 child behaviors (e.g., defiance) and a Problem Scale reflecting whether the targeted behaviors are perceived as problematic or not. In addition, we followed parents’ awareness and use of time management skills in daily life, using the Swedish Assessment of Time Management Skills (ATMS-S; (Roshanay et al., 2022). In presenting the results, we include only the ATMS-S Time Management subscale, as the others had too low internal consistencies in the current sample.

#### Analyses

Responses to open-ended treatment evaluation questions (answered in free text and/or verbally) were summarized in categories reflecting common themes, as well as feedback on the various intervention components (i.e., group sessions, individual support, adaptations and measures to facilitate participation). All analyses of differences between pre- and post-intervention reports were conducted per protocol, including complete cases, using paired-samples *t*-tests (pre vs. post). The Wilcoxon signed-rank test was used to repeat the analysis of the only markedly skewed variable (ATMS), with no change in results (data not shown). There were generally few outliers. Less than 10% of missing items on measures were treated as complete after replacing the missing item with the mean of the participant’s observed values (done only for the Skills use scale). Statistical analyses were performed in RStudio (2022.12.0 + 353).

## Results

### Participant Characteristics

#### Baseline Characteristics and Demographic Data

The mean age of the 16 participants was 41.9 (*SD* = 6). Thirteen (81.3%) were mothers. Five (31.3%) had a university degree. Nine (56.3%) were working or studying (as compared to being on sick leave, applying for work, etc.). The mean number of years since the participants received their ADHD diagnosis was 3.1 (*SD* = 2.3). Eleven (68.8%) had ADHD combined presentation; the rest had ADHD predominantly inattentive presentation or ADHD unspecified. The mean ASRS screener score was 4.2 out of 6 (*SD* = 1.6), with 11 parents (68.8%) scoring on a level corresponding to a positive ADHD screening (i.e., ≥4). Twelve parents (75%) had at least one co-existing psychiatric diagnosis (e.g., depression, anxiety, bipolar disorder, or sleep disorder). The participants’ IPSA target children (nine girls [56.3%], seven boys) had an average age of 8 years (*SD* = 2.2). Six of the target children (37.5%) had a known disability in the form of ADHD. Ten participants (62.5%) lived together with their target child full time. Eight parents (50%) lived together with the other parent of their target child, or a partner.

#### Interventions at Other Services

At baseline, all 16 parents had some form of ongoing intervention: 14 (87.5%) used pharmacotherapy to treat ADHD and/or improve mental health (75% had ADHD medication, 43.8% used antidepressants), and 11 (68.8%) participated in some kind of psychological or psychosocial intervention. At the time of follow-up assessments, two parents (12.5%) had adjusted their ongoing pharmacotherapy and two (12.5%) had received another intervention beyond that/those reported at baseline. At baseline, four of the IPSA target children (25%) had ADHD medication. At follow-up, three of them (18.8%) had received another intervention in addition to that reported at baseline, while another two (12.5%) had received a new one.

### Attrition and Data Completeness

One parent discontinued participation after the first IPSA session and was not included in further analyses. Another parent missed two baseline questionnaires (PSS, PSS-10) and was thus omitted from analyses of these scales. The mean number of complete session acceptability evaluations (administered at 14 sessions) was 9.6 (min-max = 7–12) and the mean number of homework completion ratings (administered weekly for 9 weeks) was 5 (min-max = 3–8).

### Acceptability

Ratings of overall treatment satisfaction (EQ) averaged 3.6 on a scale from 0 to 4 (*SD* = 0.4, min-max = 3–4), thus exceeding the benchmark of ≥3 out of 4. Specifically, ratings of content relevance and usefulness averaged 3.7 (*SD* = 0.3), ratings of the likelihood and confidence with which participants will use the introduced parenting skills averaged 3.6 (*SD* = 0.5), and the perceived helpfulness of exchanging experiences with other parents averaged 3.2 (*SD* = 0.9). Similarly, the session evaluations averaged 4 out of 4 (*SD* = 0.2) for the intake session, 3.9 (*SD* = 0.3) for the occupational therapist sessions, 3.7 (*SD* = 0.3) for group sessions, and 3.8 (*SD* = 0.3) for the closing session. Almost all participants (14 of 15, 93.3%) stated that they were *very likely* to recommend IPSA to a friend and gave it a final summary grade equivalent to a Swedish school grade of *pass with special distinction* (i.e., the highest).

When parents were asked to describe the ways in which IPSA had been good or helpful, there were reports of being empowered through new parenting skills (e.g., “*Gave me tools for situations where I previously had none*”) and experiences of positive change at home (e.g., “*Small things have made a huge difference, so life at home has become so much calmer,” “The atmosphere at home has become warmer and the relationship between me and my [child] feels stronger”*). Parents appreciated the combination of group and individual sessions, including group discussions (e.g., to meet other parents with ADHD, to share their advice and to take part in their descriptions of challenges similar to one’s own), and the individualized support (e.g., to break down, address, and follow up challenges individually, and allow for personal questions; “*Above all, gave support in the implementation [of skills]*”). Participants were also positive about the program approach and adaptations (e.g., “*Highlighted and clarified problem areas focusing on parents with ADHD*,” “*Helped me focus on one thing at a time*”, “*Gained focus on my needs in relation to my [child]*,” “. . . *will miss coming here and being seen and heard for who I am*”*)*, as well as the measures taken to facilitate active participation (e.g., the clear structure, SMS reminders, visual cues, videos, planning work sheets, and fidgets; “*Love the adaptation of the treatment environment*”). Moreover, parents made many suggestions on how IPSA and its material could be improved, for example by further shortening of lecture parts; by allowing more time for group discussions; by clarifying certain instructions and strategies for work tranquility; by adding booster/follow-up sessions; and by providing opportunities to participate digitally.

Pre-intervention ratings of treatment credibility and expectations for own improvement (C/EQ) averaged 44.9 out of a maximum of 54 (*SD* = 6.4).

### Active Participation

Fifteen of the 16 parents completed IPSA, that is, attended at least 9 out of 13 (group 1) or 14 (group 2) sessions. On average, completers attended 84% of sessions (*M* = 11.3, *SD* = 1.3).

In comparisons of pre- and post-intervention ratings, parents reported a more frequent use of the introduced parenting skills post intervention (Cohen’s *d* = 1.3, *p* = .003; Table 2).

**Table 2.** Pre- and Post-Intervention Self-Reports on Measures Related to Levels of Active Program Participation, Potential Harms, and Preliminary Effects, Analyzed per Protocol.

	Pre	Post	Pre vs. post		
	*M* (*SD*) [min-max]	*M* (*SD*) [min-max]	*MD* (*SEM*) [95 % CI]	*t* (*df*)	Cohen’s *d* [95 % CI]
Active participation					
Skill use	35.64 (9.07) [21–53]	44.93 (4.08) [38–53]	9.29 (2.54) [3.8, 14.77]	3.66 (13)*	1.3 [0.31, 2.29]
Performance^a^	3.5 (1.38) [2–6]	7.83 (3.06) [2–10]	4.33 (1.74) [−0.15, 8.82]	2.48 (5)	1.94 [−1.01, 4.89]
Satisfaction^a^	2.27 (2.41) [0–7]	8.18 (2.6) [2–10]	5.91 (1.35) [2.9, 8.92]	4.37 (10)*	2.36 [0.17, 4.54]
Potential harms					
PSS-10	21.21 (6.52) [11–33]	18.29 (4.32) [11–26]	−2.93 (1.76) [−6.73, 0.88]	−1.66 (13)	−0.52 [−1.21, 0.17]
HADS Anxiety	9.33 (2.94) [4–14]	9.13 (3.66) [2–14]	−0.20 (0.94) [−2.21, 1.81]	−0.21 (14)	−0.06 [−0.63. 0.51]
HADS Depression	6.27 (4.17) [1–15]	5.67 (2.69) [1–12]	−0.60 (0.90) [−2.54, 1.34]	−0.66 (14)	−0.16 [−0.67, 0.34]
Preliminary outcomes					
PSE	299.13 (78.67) [119–396]	347 (42.36) [281–421]	47.87 (15.24) [15.17, 80.56]	3.14 (14)*	0.65 [0.18, 1.12]
PSS	44.79 (9.39) [28–63]	39.64 (8.82) [29–59]	−5.14 (1.97) [−9.4, −0.89]	−2.61 (13)*	−0.56 [−1.04, −0.09]
CHAOS	41.67 (7.11) [27–53]	36.07 (6.65) [23–46]	−5.6 (2.12) [−10.15, −1.05]	−2.64 (14)*	−0.81 [−1.54, −0.09]
ECBI IS	148.27 (31.61) [104–200]	137.13 (34.45) [93–215]	−11.13 (6.95) [−26.05, 3.78]	−1.6 (14)	−0.34 [−0.78, 0.11]
ECBI PS	17.87 (7.84) [0–31]	13.93 (6.94) [5–26]	−3.93 (1.55) [−7.27, −0.6]	−2.53 (14)*	−0.53 [−0.98, −0.07]
ATMS time management	24.57 (4.78) [17–32]	27.71 (4.53) [21–34]	3.14 (1.24) [0.46, 5.82]	2.54 (14)*	0.67 [0.07, 1.28]

*Note*. ATMS = assessment of time management skills; CHAOS = Confusion, Hubbub, and Order Scale; HADS = Hospital Anxiety and Depression Scale; ECBI IS = Eyberg Child Behavior Inventory, Intensity; ECBI PS = ECBI problem: PSE = parental self-efficacy; PSS = Parental Stress Scale; PSS-10 = Perceived Stress Scale, 10-item version.

a Complete data on Performance available for second group only (*n* = 5); complete data on Satisfaction available for *n* = 11.

* Significant on the *p* < .05 level.

Parents’ between-session assignment completion ratings averaged 2 out of 4 with regard to quantity (*SD* = 0.5) and 2.2 with regard to quality (*SD* = 0.8). When summarized, results indicate that parents completed *some*, *a lot* or *all* of their assignment in 77.7% of the cases, and completed their assignment *fairly well*, *very well*, or *extremely well* in 74.8% of the cases.

Parents’ ratings of their way of managing their IPSA situation were higher post IPSA with regard to both Performance (*d* = 1.94) and Satisfaction (*d* = 2.36), although only the latter difference was statistically significant (Performance, *p* = .056; Satisfaction, *p* = .001; Table 2).

### Unintended Harm

Ten parents (62.5%) reported some kind of adverse, potentially negative, event during the study (e.g., economic difficulties, non-beneficial changes of pharmacological treatment), but none were judged to be related to participation in the program or study. There were no reports of serious adverse events. We detected no pre-to-post intervention symptom deterioration with regard to parental general stress (PSS-10), anxiety or depression (HADS; Table 2).

### Preliminary Effect-Related Outcomes

When analyzed per protocol, participants reported higher parental self-efficacy (PSE; *d* = 0.65), lower parental stress (PSS; *d* = −0.56) and lower levels of home chaos (CHAOS; *d* = −0.81) post intervention (Table 2). There was no statistically significant reduction in the frequency of child behavior problems (ECBI Intensity scale), although parents perceived their IPSA target child’s behaviors as less problematic post IPSA (ECBI Problem scale; *d* = −0.53; Table 2). In addition, parents reported better time management skills (ATMS) post intervention (*d* = 0.67; Table 2).

## Discussion

In this paper, we described the development of IPSA, a PT program for parents with ADHD. Key stakeholders (parents with ADHD) were involved across all program development phases, from the initial knowledge mobilization onwards. The results of a first test to determine whether the program prototype needed further improvement or should be further evaluated in research were promising with regard to both feasibility and preliminary effects on targeted outcomes.

### The Development of IPSA

The iterative co-creation approach used to develop IPSA was crucial to our understanding of the experiences, preferences, and needs of the IPSA target group—and thus decisive to the program design. Reassuringly, there was clear agreement between the type of adaptations proposed by parents, parenting support professionals, and the scientific community (examples in Table 1). Many have highlighted the importance of attending to the needs of the participating parent, in addition to those of their child (Smith et al., 2015). Others have theorized the potential of combining structured PT with elements to address parents’ ADHD-related needs, including factors that may affect their daily functioning and prerequisites for putting what is learned in PT into practice (Crandall et al., 2015; Johnston et al., 2012). Accordingly, IPSA was designed to combine a set of measures to facilitate PT participation and implementation, with one of the key elements being individualized occupational therapist support to help parents overcome treatment barriers and translate their newly acquired knowledge into action. Another idea aimed at further paving the way for skills practice was the targeting of one particular parent-child interaction situation in multiple ways: structurally, practically, and relationally. Essentially, this was done to enable and facilitate change that quickly makes a noticeable difference, thereby strengthening intrinsic motivation.

### Findings From the Initial Evaluation of the IPSA Program Prototype

#### Program Feasibility

Overall, this initial evaluation of the feasibility of the IPSA program prototype indicated that parents appreciated the program, participated actively in program activities, and suffered no unintended harm from participation—suggesting that IPSA was acceptable, accessible and safe. Of note, some PT trials assess parental mental health as a secondary outcome. In our context, and at this early stage, we judged it more appropriate to ensure that participation was not associated with any deterioration with regard to parental depressive symptoms or stress.

Although IPSA is comprehensive, almost all parents (15 of 16; 94%) completed the program, attending an average of 84% of the 14 regular sessions. These results are satisfactory, considering that the average attrition and attendance rates for regular PT have been estimated at 20 and 72%, respectively (Chacko et al., 2016). Moreover, while keeping in mind the uncontrolled design of the study, it can be mentioned that parents rated their use of the introduced parenting skills as more frequent post IPSA (large ES). In addition, they reported efforts to practice assigned parenting skills between sessions and appeared more satisfied with their way of managing challenging parent-child interaction situations after IPSA. Thus, although preliminary, findings add support for the potential of adaptations to facilitate active PT participation, including the use of individualized appointment reminders, efforts to prevent treatment barriers, and measures to facilitate parenting skills implementation.

Parents’ ratings of treatment satisfaction were generally high, with satisfactory ratings of content relevance and usefulness. Ratings of the perceived benefits of exchanging experiences with other parents varied slightly more, but were mainly positive. Judging from free text/oral descriptions, parents appreciated the combination of group sessions and individual support, as well as the adaptations made to increase relevance and accessibility for adults with ADHD. In addition, treatment credibility ratings were comparable to conventional PT (Nock et al., 2007).

In summary, the results regarding the feasibility of IPSA are encouraging. Importantly, however, parents also contributed valuable feedback and ideas on how the program could be further improved, to be implemented before it is evaluated in a larger study.

#### Preliminary Effect-Related Outcomes

Given the uncontrolled design of the study, these exploratory results must be interpreted with all necessary caution. However, in addition to our key findings suggesting satisfactory program feasibility, we found promising results in pre-to-post-intervention comparisons of reports of targeted outcomes such as parental self-efficacy, parenting stress and home chaos (medium-to-large ES). Moreover, parents appeared to perceive their IPSA target child’s behaviors as less problematic post IPSA, although we detected no statistically significant reduction in the frequency of child behavior problems. When proceeding with a more rigorous evaluation of IPSA and its effects in a randomized controlled trial (RCT), the ambition should be to recruit a sample large enough to detect at least medium-sized effects and allow analyses even at the subscale level; potentially enabling a more nuanced picture of which aspects of, for example, parental self-efficacy the program might in fact influence. In addition, the follow-up period should be extended to capture whether or not the potential PT effects are maintained.

### Limitations

The results of this study should be interpreted with the question of sample representativeness in mind. As in most trials addressing child or parenting interventions, fathers (19%) were underrepresented (Parent et al., 2017). The participants were self-referred and motivated to contribute views and ideas on how IPSA could be improved. They could participate without the support of an interpreter, and attend daytime sessions. However, judging by the proportion of parents with psychiatric comorbidity (75%), using ADHD medication (75%), living in a one-adult household (50%), and not currently working or studying (44%), the participants do not appear to differ significantly from those involved in clinical ADHD trials in the psychiatric outpatient care context (Hirvikoski et al., 2017), nor from adults with ADHD identified in national registry-based studies (Giacobini et al., 2023). Also, we did not confirm the participants’ ADHD diagnoses ourselves. Instead, we trusted that the clinical assessments previously made by healthcare professionals would be representative of the type of assessment future IPSA participants might be expected to have undergone, before applying for IPSA.

It should also be noted that IPSA, being a PT program, is not intended to replace other services that the parents or families need. That all participants already had some form of ongoing intervention at baseline is therefore not unexpected, although it further complicates the interpretation of our exploratory and preliminary effect-related results.

## Summary and Conclusion

The iterative co-creation approach used to develop the IPSA PT program resulted in a protocol aimed at combining the benefits of group-based PT and the flexibility of individualized PT support with ADHD adaptations, including measures to facilitate active PT participation. Overall, the results of our initial evaluation of the IPSA prototype were reassuring with regard to program feasibility—and warrant further evaluation of the IPSA program in an RCT.

## Supplemental Material

sj-pdf-1-jad-10.1177_10870547231217090 – Supplemental material for Parent Training Tailored to Parents With ADHD: Development of the Improving Parenting Skills Adult ADHD (IPSA) Program Click here for additional data file. Supplemental material, sj-pdf-1-jad-10.1177_10870547231217090 for Parent Training Tailored to Parents With ADHD: Development of the Improving Parenting Skills Adult ADHD (IPSA) Program by Therese Lindström, Sofia Buddgård, Lena Westholm, Martin Forster, Sven Bölte and Tatja Hirvikoski in Journal of Attention Disorders
