# Supplementary material for: Parent Training Tailored to Parents With ADHD: Development of the Improving Parenting Skills Adult ADHD (IPSA) Program
Source: J Atten Disord. 2023 Dec 28;28(4):531–41. doi: 10.1177/10870547231217090 (PMC10838472; doi:10.1177/10870547231217090)
Supplement: sj-pdf-1-jad-10.1177_10870547231217090 – Supplemental material for Parent Training Tailored to Parents With ADHD: Development of the Improving Parenting Skills Adult ADHD (IPSA) Program [file sj-pdf-1-jad-10.1177_10870547231217090.pdf]

Supplemental Material/Appendices

for "Parent training tailored to parents with ADHD: Development of the Improving Parenting Skills Adult ADHD (IPSA) program"

by Therese Lindström, Sofia Buddgård, Lena Westholm, Martin Forster,

Sven Bölte, & Tatja Hirvikoski

Including:

Supplementary Table 1

Supplementary Table 2

Supplementary Table 3

Supplementary Table 1 Overview of the three-phase program development process

Phase (year)	Activity	Outcome
1 (2016)	Mapping of existing interventions and needs, including:<br>- a systematic literature review,<br>- stakeholder interviews (involving parents and professionals).	Report (Swedish only)
2 (2017)	Development of program prototype, based on:<br>- evidence-based PT interventions (Swedish and international),<br>- interviews/workshops with key stakeholders (i.e., parents),<br>- scientific and clinical literature in relevant areas.	Program prototype
3 (2018)	Evaluation and refinement of the prototype, based on:<br>- a before-after study of feasibility and preliminary outcomes [a],<br>- parent program evaluations and feedback.	Refined program, ready for further evaluation

[a] Results reported in the article

Supplementary Table 2 Themes and content of the IPSA group sessions, individual intake session and individual closing session

Session theme (length in minutes)	Session content	Parenting skill to practice between sessions
Individual intake session: Introduction (90)	Information to ensure realistic expectations. Brief clinical assessment to identify parental needs, areas for development, hopes for change, etc. Work to choose and define an IPSA situation[a] and to formulate individual goal(s). Identification of, and problem solving with respect to, potential treatment barriers.	
Group session 1: Parental ADHD in everyday family life (150)	Introduction to: group session procedures and materials; the manifestation and impact of adult ADHD symptoms and executive function impairments in parenting and everyday family life, including the IPSA situation; the importance of building on what works, of finding one's way of doing things and managing challenges.	During the session, parents share their best life hacks[b]; those who feel inspired are encouraged to try one, while the others continue their individual work to improve their prerequisites and develop their techniques to manage their IPSA situation.
Group session 2: Strengthen your parental prerequisites (150)	Introduction to: the importance of considering one's own prerequisites (e.g., levels of stress, energy, overall vulnerability) for managing challenging family/parent-child situations; ways to strengthen/improve one's own parental prerequisites; procedures for detailed planning of the week's homework (i.e., skills-practice).	Micropause: Improve your own prerequisites for managing challenging parent-child interaction situations (e.g., your IPSA situation) by taking a micro pause right before (to wind down, recharge, slow racing thoughts, breath, find presence, etc.).
Group session 3: Strengthen the parent-child relationship (150)	Introduction to: positive reinforcement, labeled praise, and contingent rewards; conscious allocation of parental attention; the importance and power of moments reserved for positive and undemanding parent-child interaction and togetherness (in many contexts referred to as parent-child quality time).	Seize the moment: Strengthen the parent-child relationship by filling everyday life with more positive parent-child interaction and active togetherness, concentrated in short moments (e.g., three minutes at a time), preferably in everyday situations.
Group session 4: Facilitate cooperation (150)	Introduction to: the importance of considering the child's prerequisites (e.g., levels of energy) when requesting cooperation; preparations before (transitions between) activities; ways to give effective commands, including strategies to give the child time to act before repeating the command.	Effective commands: Facilitate cooperation by giving prompts and commands in an effective way (e.g., clear, concise; given one at a time, when in direct contact with the child; using labeled praise and withholding unnecessary repeats).
Group session 5: Act calmly on the outside (150)	Introduction to: active listening; emotional reactions; how parents can reduce the risk of parent-child conflict and emotional escalation by regulating their own emotional expression and trying to act calmly on the outside, even when there are strong emotions inside; factors that may increase/decrease vulnerability to strong reactions (e.g., stress); a variety of methods that can make it easier to calm down.	Act calmly on the outside (regardless of the feelings inside): Reduce the risk of emotional escalation by following a well-rehearsed strategy to increase your chances of acting calmly: stop, breath, do what you need (any method) to be able to speak in a calm way, and use a relaxed body language.
Group session 6: Choose your battles (150)	Introduction to: ways to be more consistent and predictable; ways to consciously choose which issues are important to stand your ground on or enforce – and which can be let go. Summary of previous sessions, skills and take-home messages.	Choose your battles wisely: Reduce the risk of conflict by consistently choosing not to act on (e.g., correct, point out, nag, reprimand, demand) certain minor issues, misbehaviors.
Individual closing session: Summary (60)	Summary of the IPSA period and activities, including steps to evaluate progress, highlight changes and improvements, identify any remaining needs, and formulate a plan to maintain treatment gains.	
Booster group session (150)	Summary of skills and take-home messages, as well as what the parents wish to continue to do, or remind themselves about, going forward.	

[a] IPSA situation = a particularly challenging parent-child interaction situation that the parent wants to work to manage in a better way
[b] Life hack = simple, smart, or creative tips, shortcuts or solutions that make specific family situations a little easier

Supplementary Table 3 Information on response scales, score ranges, interpretations, and internal consistencies of the self-report questionnaires used in the study

	Response scale	Min-max scores	Interpretation (better rating) [a]	α [b]
**Parental ADHD**				
ASRS	0 (never) - 4 (very often)	0-6 [c]	Lower	.72
**Acceptability**				
Treatment satisfaction	0 (not at all) - 4 (a great deal)	0-4	Higher	.69
C/EQ	1 (not at all) - 9 (very much)<br>and 0% - 100 % (later recoded to 1-9)	6-54	Higher	.86
**Active participation**				
Homework Quantity	0 (none) - 4 (all)	0-4	Higher	-
Homework Quality	0 (not at all well) - 4 (extremely well)	0-4	Higher	-
Skill use	0 (never) - 6 (very often, can be several times a day)	0-66	Higher	.83
Management of IPSA situation: Performance	0 (not at all good) - 10 (very good)	0-10	Higher	-
Management of IPSA situation: Satisfaction	0 (not at all satisfied) - 10 (very satisfied)	0-10	Higher	-
**Potential harms**				
PSS-10	0 (never) - 4 (very often)	0-40	Lower	.89
HADS Anxiety	0 - 3 (anchors vary)	0-21	Lower	.69
HADS Depression	0 - 3 (anchors vary)	0-21	Lower	.87
**Preliminary outcomes**				
PSE	0 (completely disagree) - 10 (totally agree)	0-480	Higher	.97
PSS	1 (strongly disagree) - 5 (strongly agree)	18-90	Lower	.82
CHAOS	1 (not at all) - 4 (very much)	15-60	Lower	.82
ECBI IS	1 (never) - 7 (always)	36-252	Lower	.92
ECBI PS	0 (No) or 1 (Yes)	0-36	Lower	.89
ATMS Time management	1 (never) - 4 (always)	11-44	Higher	.80

Note. ASRS = Adult ADHD Self-Report Scale; ATMS = Assessment of Time Management Skills; C/EQ = Credibility Expectancy Questionnaire; CHAOS = Confusion, Hubbub, and Order Scale; ECBI IS = Eyberg Child Behavior Inventory, Intensity; ECBI PS = ECBI Problem scale; HADS = Hospital Anxiety and Depression Scale; PSE = Parental Self-Efficacy scale; PSS = Parental Stress Scale; PSS-10 = Perceived Stress Scale, 10-item version.

[a] States whether a higher or a lower score is "better", that is, which type of score (a higher or a lower) indicates the desired or preferred level of the rated construct

[b] Cronbach's alpha for all scales except for 1-item scales (na) and scales with dichotomous response scales (for which Kuder-Richardson 20 formula is used)

[c] When the scale is used for screening purposes, a score of ≥ 4 indicates a positive screening for adult ADHD
